# Prescribing Patterns of SGLT2 Inhibitors and GLP‐1 Receptor Agonists in Patients With T2DM and ASCVD in South Korea

**DOI:** 10.1002/pds.70183

**Published:** 2025-06-30

**Authors:** Yeong Rok Eom, Hajung Joo, Seung Eun Chae, Nam Kyung Je

**Affiliations:** ^1^ College of Pharmacy Pusan National University Busan Republic of Korea; ^2^ College of Pharmacy Northeastern University Boston USA; ^3^ Research Institute for Drug Development Pusan National University Busan Republic of Korea

**Keywords:** atherosclerotic cardiovascular disease, glucagon‐like peptide‐1 receptor agonists, sodium‐glucose cotransporter 2 inhibitors, type 2 diabetes mellitus

## Abstract

**Background:**

Despite the cardiovascular benefits of sodium‐glucose cotransporter 2 inhibitors (SGLT2i) and glucagon‐like peptide‐1 receptor agonists (GLP1RA) in patients with type 2 diabetes mellitus (T2DM) and atherosclerotic cardiovascular disease (ASCVD), their utilization remains low globally. This study aimed to evaluate the utilization of SGLT2i and GLP1RA in patients with T2DM and ASCVD, as well as the factors associated with their use in South Korea.

**Methods:**

We conducted a retrospective study using the National Patient Sample claims data from 2015 to 2020. Adults aged 20 years or older with confirmed diagnoses of both T2DM and ASCVD between March 1 and October 31 of each year were included. The utilization of SGLT2i and GLP1RA was assessed based on prescriptions filled within 60 days of the index date. Multivariable logistic regression was used to identify factors associated with their use. Annual trends in utilization were evaluated using the Cochran–Armitage trend test.

**Results:**

In our study of 57 576 study population, the use of SGLT2i increased from 1.20% in 2015 to 10.51% by 2020. GLP1RA usage increased from 0% to 1.17% over the same period. Older age, chronic kidney disease (OR 0.52, 95% CI 0.41–0.66), and concurrent use of dipeptidyl peptidase 4 inhibitors (DPP4i) (OR 0.09, 95% CI 0.09–0.10) significantly reduced the likelihood of SGLT2i use. In contrast, factors such as comorbid dyslipidemia (OR 1.41, 95% CI 1.25–1.60), heart failure (OR 1.22, 95% CI 1.09–1.37), concurrent use of sulfonylurea (SU) (OR 1.30, 95% CI 1.20–1.40), and prescriptions from cardiologists (OR 1.22, 95% CI 1.07–1.40) were positively associated with higher SGLT2i usage. For GLP1RA, negative influences included older age, concurrent DPP4i use (OR 0.12, 95% CI 0.08–0.16), and non‐endocrinologist prescription, whereas female sex (OR 1.35, 95% CI 1.06–1.73), dyslipidemia (OR 1.68, 95% CI 1.10–2.66), and the use of insulin (OR 3.71, 95% CI 2.83–4.85), or SU (OR 3.13, 95% CI 2.44–4.02) use were positive factors.

**Conclusions:**

Despite the known cardiovascular benefits and increasing utilization trends of SGLT2i and GLP1RA, our findings reveal that 88.35% of eligible patients with T2DM and ASCVD remained untreated with these agents as of 2020. This study suggests disparities in the use of these agents based on patients' characteristics and physician specialties. Further efforts to explore and address potential barriers to the use of these agents could enhance their clinical benefits by improving access for high‐risk patients.

**Summary**

- Between 2015 and 2020, the use of SGLT2 inhibitors and GLP‐1 receptor agonists increased among Korean patients with type 2 diabetes and ASCVD, but overall prescription rates remained low, with only 11.7% receiving either agent by 2020.
- Prescribing patterns were significantly influenced by patient characteristics such as age and comorbidities (e.g., CKD, heart failure), as well as by concurrent use of other antidiabetic medications.
- Physician specialty also affected prescribing: cardiologists were more likely to prescribe SGLT2 inhibitors, while endocrinologists favored GLP‐1 receptor agonists.
- Restrictive reimbursement criteria during the study period likely contributed to underuse. Recent expansions in coverage may improve access to these cardioprotective therapies.

## 1 Introduction

Type 2 diabetes mellitus (T2DM) is a common chronic condition affecting millions of people worldwide [1] and the prevalence of T2DM continues to increase [2]. T2DM is an important risk factor for atherosclerotic cardiovascular disease (ASCVD), making it imperative to effectively address its associated risks [3, 4]. Particularly, individuals with both T2DM and ASCVD have higher risk for cardiovascular disease (CVD) events and related mortality than those with T2DM alone, highlighting the importance of proactive CVD management and prevention in this particular population [5].

In recent years, several large clinical trials have been conducted to examine the cardiovascular risk reduction benefits of various antidiabetic agents [6, 7, 8, 9, 10, 11, 12]. Among these agents, sodium‐glucose cotransporter 2 inhibitors (SGLT2i) and glucagon‐like peptide‐1 receptor agonists (GLP1RA) have demonstrated cardiovascular protective effects [6, 7, 8, 10, 11, 12]. In individuals with T2DM, SGLT2i and GLP1RA have been shown to reduce major adverse cardiovascular events (MACE) by 11% and 12%, respectively [13, 14]. Moreover, these agents have also demonstrated a substantial reduction in MACE, with a 14% reduction for SGLT2i and a 13% reduction for GLP1RA in individuals with concurrent ASCVD [13, 14]. The accumulating evidence from these trials had led to a paradigm shift in the utilization of these agents. As a result, the updated guidelines of the American Diabetes Association (ADA), European Association for the Study of Diabetes (EASD), and Korean Diabetes Association (KDA) recommend the use of SGLT2i and GLP1RA in patients with T2DM and ASCVD to mitigate cardiovascular risk [15, 16, 17].

Despite these updated guidelines, global data have indicated insufficient utilization of SGLT2i and GLP1RA [18, 19, 20, 21, 22, 23, 24, 25]. A nationwide study in the United States showed that the utilization rate of SGLT2i and GLP1RA increased between 2018 and 2021 in patients with T2DM and ASCVD; however, these agents remained underused in comparison with other antidiabetic agents, such as sulfonylureas (SU) and dipeptidyl‐peptidase 4 inhibitors (DPP4i) [19]. Similarly, a study conducted in the United Kingdom revealed an increase in the utilization rate of SGLT2i and GLP1RA in patients with T2DM and CVD; nevertheless, these agents were less commonly prescribed to patients with concomitant CVD than to those without CVD [20]. A Canadian regionwide study demonstrated increased use of SGLT2i and GLP1RA, but their utilization for cardioprotection still falls behind well‐established secondary prevention agents like statins and angiotensin‐converting enzyme inhibitors/angiotensin receptor antagonists (ACEi/ARB) [21].

With the publication of several large clinical trials and changes in diabetes guidelines, considerable changes in prescription patterns and utilization of SGLT2i and GLP1RA for patients with T2DM and ASCVD were expected in South Korea. However, only a few studies have been conducted on this topic in South Korea [26]. Therefore, our study aimed to evaluate the yearly prescription trend for antidiabetic agents, including SGLT2i and GLP1RA, in patients with T2DM and ASCVD. In addition, we compared the characteristics of patients who received these medications with those who did not and examined the factors influencing their use.

## 2 Methods

### 2.1 Data Sources

We conducted a retrospective cross‐sectional study using National Patient Sample data collected by the Korean Health Insurance Review and Assessment Service (HIRA‐NPS) from 2015 to 2020. The HIRA‐NPS represents 2%–3% of the claims data for the Korean population, consistently 3% prior to 2019 [27]. Given the 2%–3% annual stratified random sampling design, the likelihood of including the same patient in multiple years is expected to be minimal; however, the extent of potential overlap cannot be confirmed due to the nature of the dataset.

The Korean health insurance system is categorized into three groups: National Health Insurance (NHI), Medical Aid (MedAid), and Patriots and Veterans Insurance (PVI). The majority of the Korean population is covered by NHI (97.2%), with the remaining 3% covered by MedAid or PVI [28]. As HIRA collects data for reimbursement under the NHI, the HIRA dataset encompasses a comprehensive view of healthcare services provided across South Korea. The HIRA‐NPS is a representative sample of this dataset, reflecting healthcare utilization patterns under the NHI system [27].

### 2.2 Study Population

Our study extracted data on adult patients aged ≥ 20 years with concurrent T2DM and ASCVD confirmed between March 1 and October 31 each year who were also prescribed at least one antidiabetic agent.

Patients with T2DM were identified using the Korean Classification of Disease 7th edition (KCD‐7) code E11, either as the main diagnosis or a subdiagnosis. We excluded those solely coded with E14 (unspecified diabetes mellitus) to ensure specificity in the identification of confirmed T2DM. Given the frequent overlap in coding practices in Korea, we believe that the exclusion of E14 is unlikely to have significantly impacted the representativeness of the T2DM population in this study. Patients aged < 20 years were excluded from the study. The presence of ASCVD was determined using both KCD‐7 and procedure codes (Table S1). ASCVD was defined in accordance with the guidelines of the American College of Cardiology and American Heart Association (ACC/AHA) 2013, encompassing ischemic heart diseases, such as myocardial infarction and angina, peripheral artery diseases, ischemic stroke, transient ischemic attack (TIA), and arterial revascularization procedures, including coronary interventions [29]. Due to the nature of HIRA‐NPS data, it does not specify whether T2DM and ASCVD were newly diagnosed in that year or had been identified in previous years. Therefore, we included patients with confirmed coexistence of these conditions each specified year, regardless of whether they were newly diagnosed or existing cases. We evaluated all prescriptions within 60 days of the index date (the date when concurrent T2DM and ASCVD were initially identified) and included patients who were prescribed at least one antidiabetic agent as the final study population (Table S2).

We collected demographic information for the study population, including age, sex, insurance type, region, type of medical institution, and physician's specialty. Ages were stratified into five categories: 20–39, 40–49, 50–59, 60–69, and ≥ 70 years. Insurance types were divided into the NHI and MedAid/PVI groups. Medical institutions were grouped into five categories: tertiary hospitals, general hospitals, hospitals, clinics, and others. Regions were classified into three groups: capital city, metropolitan cities, and others. Physician specialties were categorized into five groups: cardiologists, endocrinologists, nephrologists, other internists (internists specializing in neither cardiology, endocrinology, nor nephrology), and other physicians.

Patients' comorbidities were identified based on the identification of KCD‐7 codes for additional diagnoses within 60 days preceding the index date, as detailed in Table S3. The use of other cardioprotective agents was determined based on prescriptions for statins or ACEi/ARB.

### 2.3 Outcome

The outcome of our study was the utilization of SGLT2i or GLP1RA among patients with concurrently diagnosed T2DM and ASCVD. Patients prescribed dapagliflozin, empagliflozin, ipragliflozin, or ertugliflozin were classified as SGLT2i users. Similarly, those prescribed dulaglutide, exenatide, lixisenatide, or albiglutide were classified as GLP1RA users.

### 2.4 Statistical Analysis

Our analysis commenced with a characterization of the study population, identifying patients who were prescribed either SGLT2i or GLP1RA. Descriptive statistics including frequency percentages were used to summarize demographic and clinical features, and the chi‐square test was conducted to compare the characteristics of users versus nonusers of these medications. To explore factors influencing the utilization of SGLT2i and GLP1RA, we conducted multivariable logistic regression analysis.

For trend analysis, we assessed the yearly utilization patterns of SGLT2i and GLP1RA from 2015 to 2020. This included evaluating the combined use trends of either SGLT2i or GLP1RA, as well as analyzing the specific trends for each SGLT2i agent over the same period. The Cochran–Armitage trend test was employed to verify the statistical significance of these trends providing insights into how prescribing patterns have evolved annually.

We performed all analyses using the R Statistical Software (version 3.5.1; R Foundation for Statistical Computing, Vienna, Austria), and the level of significance was set at *p* < 0.05.

## 3 Results

### 3.1 Characteristic of the Study Population

**FIGURE 1:**
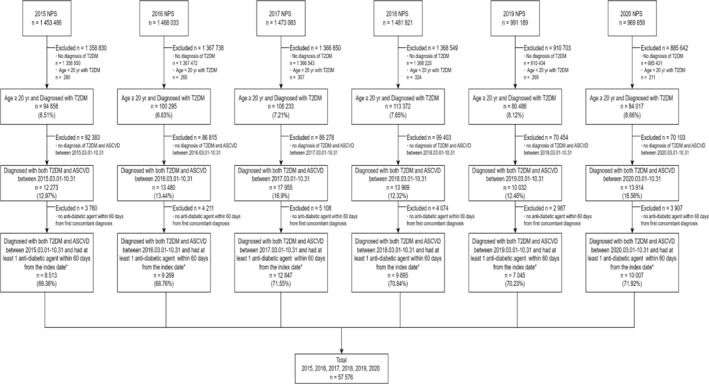
Selection of the study population. * Date when concurrent T2DM and ASCVD were initially identified; T2DM: Type 2 diabetes mellitus; ASCVD, atherosclerotic cardiovascular disease; NPS: National patient Sample data.

**FIGURE 2:**
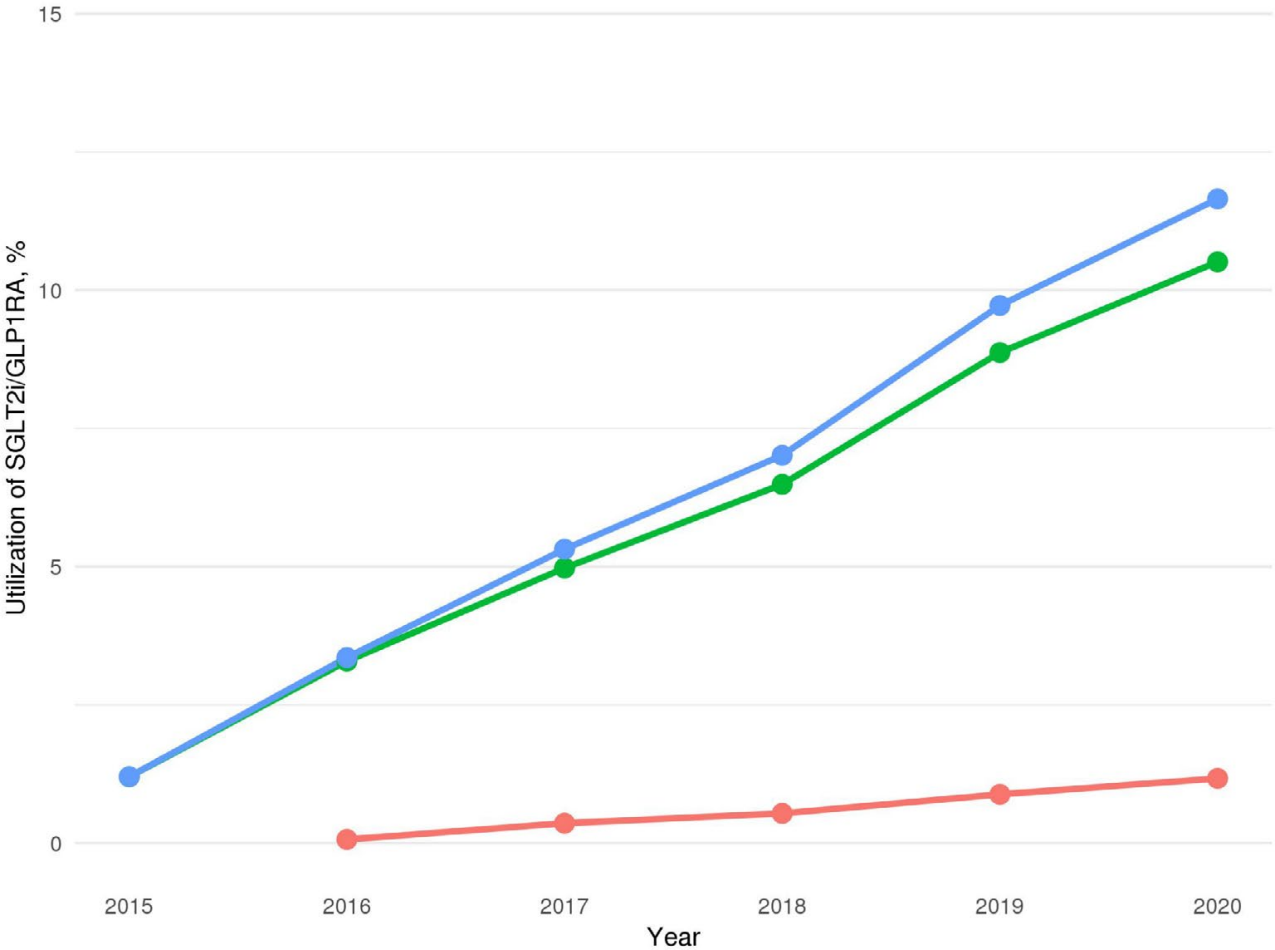
The utilization rate of SGLT2i and GLP1RA between 2015 and 2020. Lines represent the proportion of patients prescribed each medication class: SGLT2i (green), GLP1RA (red), and either agent (blue). The Cochran‐Armitage trend test showed a significant upward trend for all three categories (*p* < 0.001). SGLT2i: Sodium‐glucose cotransporter 2 inhibitors; GLP1RA: Glucagon‐like peptide‐1 receptor agonists.

Between 2015 and 2020, 57 576 patients with T2DM and ASCVD who were prescribed at least one antidiabetic agent were selected (Figure 1). The utilization of SGLT2i increased more than 8‐fold during the study period, from 1.20% in 2015 to 10.51% in 2020 (Figure 2). In contrast, the utilization rate of GLP1RA increased marginally from 0% in 2015 to 1.17% in 2020. By 2020, the proportion of patients treated with either of these agents reached 11.65%.

In terms of the utilization of oral antidiabetic agents over the years, metformin consistently remained the most used drug. From 2015 to 2019, the top four oral antidiabetic agents used remained unchanged: metformin > DPP4i > SU> thiazolidinediones (TZD) (Figure S1). However, in 2020, the utilization of SGLT2i (10.51%) slightly surpassed that of TZD (10.15%) (Table S4). Among noncardioprotective antidiabetic agents, the use of DPP4i and TZD exhibited an increasing trend, whereas the use of SU showed a decreasing trend.

The overall utilization rate of SGLT2i was 5.84% (*n* = 3365) (Table 1). The use of SGLT2i decreased with age, reaching the lowest value among those aged ≥ 70 years (2.76%). Women had a lower utilization rate of SGLT2i compared to men. Regarding comorbidities, SGLT2i use was higher in patients with heart failure and dyslipidemia compared to those without these conditions. In contrast, patients with chronic kidney disease had lower SGLT2i utilization than those without. Patients prescribed other cardioprotective agents, such as statins and ACEi/ARB, had higher rates of SGLT2i use than those who were not prescribed these medications. Patients using DPP4i had significantly lower SGLT2i utilization compared to nonusers (1.48% vs. 11.87%), whereas individuals using SU had higher utilization rates (6.19% vs. 5.61%). In terms of physician specialty, cardiologists had the highest SGLT2i utilization (9.65%), followed by endocrinologists, other internists, nephrologists, and other physicians.

**TABLE 1:** Characteristics of the study population categorized based on the use of SGLT2i or GLP1RA.

		SGLT2i			GLP1RA			SGLT2i or GLP1RA		
	*N* (%)	Nonusers (%)	Users (%)	*p*	Nonusers (%)	Users (%)	*p*	Nonusers (%)	Users (%)	*p*
Total	57 576	54 211 (94.16)	3365 (5.84)		57 292 (99.51)	284 (0.49)		53 935 (93.68)	3641 (6.32)	
Age (years)				< 0.001			< 0.001			< 0.001
20–39	635 (1.10)	523 (82.36)	112 (17.64)		620 (97.64)	15 (2.36)		508 (80.00)	127 (20.00)	
40–49	3273 (5.69)	2851 (87.11)	422 (12.89)		3229 (98.66)	44 (1.34)		2809 (85.82)	464 (14.18)	
50–59	11 316 (19.65)	10 221 (90.32)	1095 (9.68)		11 231 (99.25)	85 (0.75)		10 138 (89.59)	1178 (10.41)	
60–69	17 611 (30.59)	16 558 (94.02)	1053 (5.98)		17 528 (99.53)	83 (0.47)		16 477 (93.56)	1134 (6.44)	
≥ 70	24 741 (42.97)	24 058 (97.24)	683 (2.76)		24 684 (99.77)	57 (0.23)		24 003 (97.02)	738 (2.98)	
Sex				< 0.001			0.700			< 0.001
Male	30 861 (53.60)	28 888 (93.61)	1973 (6.39)		30 712 (99.52)	149 (0.48)		28 741 (93.13)	2120 (6.87)	
Female	26 715 (46.40)	25 323 (94.79)	1392 (5.21)		26 580 (99.50)	135 (0.50)		25 194 (94.31)	1521 (5.69)	
Institution				< 0.001			< 0.001			< 0.001
Tertiary	9854 (17.12)	9129 (92.64)	725 (7.36)		9788 (99.33)	66 (0.67)		9064 (91.98)	790 (8.02)	
General	15 345 (26.65)	14 337 (93.43)	1008 (6.57)		15 240 (99.32)	105 (0.68)		14 234 (92.76)	1111 (7.24)	
Hospital	3950 (6.86)	3760 (95.19)	190 (4.81)		3934 (99.60)	16 (0.40)		3746 (94.84)	204 (5.17)	
Clinic	27 146 (47.15)	25 732 (94.79)	1414 (5.21)		27 049 (99.64)	97 (0.36)		25 638 (94.45)	1508 (5.55)	
Others	1281 (2.22)	1253 (97.81)	28 (2.19)		1281 (100)	0 (0)		1253 (97.81)	28 (2.19)	
Insurance				0.002			0.419			0.006
NHI	52 290 (90.82)	49 183 (94.06)	3107 (5.94)		52 036 (99.51)	254 (0.49)		48 937 (93.59)	3353 (6.41)	
MedAid/PVI	5286 (9.18)	5028 (95.12)	258 (4.88)		5256 (99.43)	30 (0.57)		4998 (94.55)	288 (5.45)	
Region				0.004			< 0.001			< 0.001
Capital	13 375 (23.23)	12 536 (93.73)	839 (6.27)		13 282 (99.31)	93 (0.69)		12 445 (93.05)	930 (6.95)	
Metropolitans	14 673 (25.49)	13 782 (93.93)	891 (6.07)		14 606 (99.54)	67 (0.46)		13 717 (93.49)	956 (6.51)	
Others	29 528 (51.28)	27 893 (94.46)	1635 (5.54)		29 404 (99.58)	124 (0.42)		27 773 (94.06)	1755 (5.94)	
Dyslipidemia				< 0.001			< 0.001			< 0.001
No	11 789 (20.48)	11 348 (96.26)	441 (3.74)		11 761 (99.76)	28 (0.24)		11 320 (96.02)	469 (3.98)	
Yes	45 787 (79.52)	42 863 (93.61)	2924 (6.39)		45 531 (99.44)	256 (0.56)		42 615 (93.07)	3172 (6.93)	
Heart failure				0.012			0.299			0.007
No	50 432 (87.59)	47 531 (94.25)	2901 (5.75)		50 189 (99.52)	243 (0.48)		47 295 (93.78)	3137 (6.22)	
Yes	7144 (12.41)	6680 (93.51)	464 (6.49)		7103 (99.43)	41 (0.57)		6640 (92.95)	504 (7.05)	
CKD				< 0.001			0.040			< 0.001
No	54 490 (94.64)	51 207 (93.98)	3283 (6.02)		54 229 (99.52)	261 (0.48)		50 954 (93.51)	3536 (6.49)	
Yes	3086 (5.36)	3004 (97.34)	82 (2.66)		3063 (99.26)	23 (0.74)		2981 (96.60)	105 (3.40)	
Hypertension				0.128			0.427			0.076
No	14 438 (25.08)	13 557 (93.90)	881 (6.10)		14 361 (99.47)	77 (0.53)		13 480 (93.37)	958 (6.63)	
Yes	43 138 (74.92)	40 654 (94.24)	2484 (5.76)		42 931 (99.52)	207 (0.48)		40 455 (93.78)	2683 (6.22)	
ACEi/ARB				0.006			0.981			0.010
No	26 315 (45.71)	24 854 (94.45)	1461 (5.55)		26 185 (99.51)	130 (0.49)		24 726 (93.96)	1589 (6.04)	
Yes	31 261 (54.29)	29 357 (93.91)	1904 (6.09)		31 107 (99.51)	154 (0.49)		29 209 (93.44)	2052 (6.56)	
Statin				< 0.001			< 0.001			< 0.001
No	21 130 (36.70)	20 212 (95.66)	918 (4.34)		21 058 (96.66)	72 (0.34)		20 140 (95.32)	990 (4.68)	
Yes	36 446 (63.30)	33 999 (93.29)	2447 (6.71)		36 234 (99.42)	212 (0.58)		33 795 (92.73)	2651 (7.27)	
DPP4i				< 0.001			< 0.001			< 0.001
No	24 172 (41.98)	21 302 (88.13)	2870 (11.87)		23 928 (98.99)	244 (1.01)		21 065 (87.15)	3107 (12.85)	
Yes	33 404 (58.02)	32 909 (98.52)	495 (1.48)		33 364 (99.88)	40 (0.12)		32 870 (98.40)	534 (1.60)	
SU				0.004			< 0.001			< 0.001
No	34 111 (59.25)	32 198 (94.39)	1913 (5.61)		33 991 (99.65)	120 (0.35)		32 080 (94.05)	2031 (5.95)	
Yes	23 465 (40.75)	22 013 (93.81)	1452 (6.19)		23 301 (99.30)	164 (0.70)		21 855 (93.14)	1610 (6.86)	
Insulin				0.109			< 0.001			0.006
No	50 976 (88.54)	47 968 (94.10)	3008 (5.90)		50 807 (99.67)	169 (0.33)		47 804 (93.78)	3172 (6.22)	
Yes	6600 (11.46)	6243 (94.59)	357 (5.41)		6485 (98.26)	115 (1.74)		6131 (92.89)	469 (1.11)	
Physician specialty				< 0.001			< 0.001			< 0.001
Cardiologists	4498 (7.81)	4064 (90.35)	434 (9.65)		4491 (99.84)	7 (0.16)		4057 (90.20)	441 (9.80)	
Endocrinologists	13 213 (22.95)	12 266 (92.83)	947 (7.17)		13 080 (98.99)	133 (1.01)		12 135 (91.84)	1078 (8.16)	
Nephrologists	1287 (2.23)	1236 (96.04)	51 (3.96)		1277 (99.22)	10 (0.78)		1226 (95.26)	61 (4.74)	
Other internists	25 009 (43.44)	23 612 (94.41)	1397 (5.59)		24 903 (99.58)	106 (0.42)		23 511 (94.01)	1498 (5.99)	
Other physicians	13 569 (23.57)	13 033 (96.05)	536 (3.95)		13 541 (99.79)	28 (0.21)		13 006 (95.85)	563 (4.15)	

Abbreviations: ACEi/ARB, angiotensin‐converting enzyme inhibitors/angiotensin receptor antagonists; CKD, chronic kidney disease; DPP4i, dipeptidyl‐peptidase 4 inhibitors; GLP1RA, glucagon‐like peptide‐1 receptor agonists; MedAid, Medical Aid; NHI, National Health Insurance; PVI, Patriots and Veterans Insurance; SGLT2i, sodium‐glucose cotransporter 2 inhibitors; SU, sulfonylureas.

The overall utilization rate of GLP1RA was 0.49% (*n* = 284). GLP1RA utilization decreased with age. Patients with dyslipidemia or chronic kidney disease had higher GLP1RA utilization than those without these conditions. Patients treated with statins had higher GLP1RA use than those not treated with statins. Patients using DPP4i had a lower utilization rate of GLP1RA compared to nonusers (0.12% vs. 1.01%), whereas those using SU had higher utilization rates compared to nonusers (0.70% vs. 0.35%). Additionally, patients using insulin had significantly higher GLP1RA use compared to nonusers (1.74% vs. 0.33%). Finally, endocrinologists had the highest GLP1RA use (1.01%), followed by nephrologists, other internists, other physicians, and cardiologists.

### 3.2 Factors Associated With the Use of SGLT2i and GLP1RA

The results of the multivariable logistic regression analysis are presented in Table 2. Advanced age was associated with reduced SGLT2i use. Among the comorbidities, heart failure and dyslipidemia had a positive influence on SGLT2i use. In contrast, chronic kidney disease was associated with the underutilization of SGLT2i. In terms of physician specialty, cardiologists were more likely to prescribe SGLT2i to patients with T2DM and ASCVD (OR = 1.22, [1.07, 1.40]) than were endocrinologists. In contrast, other internists and physicians were less likely to prescribe SGLT2i than were endocrinologists. Patients taking statins and ACEi/ARB were more likely to use SGLT2i than those not taking these medications. DPP4i use was a strong negative correlate SGLT2i use (OR = 0.09, [0.09, 0.10]), whereas SU use was associated with increased SGLT2i use (OR = 1.30, [1.20, 1.40]).

**TABLE 2:** Factors influencing the use of SGLT2i and GLP1RA.

	SGLT2i use		GLP1RA use		SGLT2i or GLP1RA use	
	Adj. OR	95% CI	Adj. OR	95% CI	Adj. OR	95% CI
Age (years)						
20–30 (R)	1		1		1	
40–49	0.73	0.57–0.93	0.74	0.41–1.41	0.70	0.55–0.90
50–59	0.48	0.38–0.60	0.40	0.23–0.74	0.44	0.36–0.56
60–69	0.26	0.21–0.33	0.24	0.14–0.44	0.24	0.20–0.31
≥ 70	0.12	0.09–0.15	0.12	0.07–0.23	0.11	0.09–0.13
Sex						
Male (R)	1		1		1	
Female	1.02	0.95–1.10	1.35	1.06–1.73	1.05	0.97–1.13
Institution						
Tertiary (R)	1		1		1	
General	0.98	0.87–1.09	1.23	0.89–1.71	1.00	0.90–1.11
Hospital	0.88	0.71–1.08	1.35	0.66–2.7	0.90	0.73–1.11
Clinic	0.89	0.76–1.04	1.21	0.71–2.12	0.91	0.78–1.06
Others	0.51	0.33–0.76	0	0	0.50	0.32–0.74
Insurance						
NHI (R)	1		1		1	
MedAid/PVI	0.88	0.76–1.00	1.04	0.69–1.51	0.89	0.78–1.01
Region						
Capital (R)	1		1		1	
Metropolitans	1.04	0.94–1.16	0.80	0.57–1.11	1.02	0.92–1.13
Others	1.02	0.92–1.12	0.78	0.59–1.05	0.99	0.90–1.09
Dyslipidemia						
No (R)	1		1		1	
Yes	1.41	1.25–1.60	1.68	1.10–2.66	1.44	1.28–1.62
Heart failure						
No (R)	1		1		1	
Yes	1.22	1.09–1.37	1.32	0.92–1.85	1.24	1.11–1.38
CKD						
No (R)	1		1		1	
Yes	0.52	0.41–0.66	1.05	0.63–1.69	0.57	0.46–0.71
Hypertension						
No (R)	1		1		1	
Yes	0.96	0.87–1.07	0.94	0.67–1.32	0.95	0.86–1.06
ACEi/ARB						
No (R)	1		1		1	
Yes	1.13	1.03–1.24	1.02	0.76–1.39	1.13	1.03–1.23
Statin						
No (R)	1		1		1	
Yes	1.42	1.29–1.57	1.63	1.20–2.24	1.45	1.33–1.60
DPP4i						
No (R)	1		1		1	
Yes	0.09	0.09–0.10	0.12	0.08–0.16	0.09	0.08–0.10
SU						
No (R)	1		1		1	
Yes	1.30	1.20–1.40	3.13	2.44–4.02	1.41	1.31–1.51
Insulin						
No (R)	1		1		1	
Yes	0.63	0.56–0.71	3.71	2.83–4.85	0.80	0.71–0.90
Physician specialty						
Endocrinologists (R)	1		1		1	
Cardiologists	1.22	1.07–1.40	0.24	0.10–0.49	1.13	0.99–1.29
Nephrologists	0.85	0.61–1.15	0.80	0.37–1.58	0.84	0.62–1.12
Other internists	0.81	0.70–0.94	0.54	0.33–0.86	0.77	0.67–0.89
Other physicians	0.55	0.46–0.64	0.31	0.17–0.55	0.51	0.44–0.60
C statistic	0.82		0.88		0.82	
*p* of H‐L test	< 0.001		0.229		< 0.001	

Abbreviations: ACEi/ARB, angiotensin‐converting enzyme inhibitors/angiotensin receptor antagonists; CKD, chronic kidney disease; DPP4i, dipeptidyl‐peptidase 4 inhibitors; SU, sulfonylureas; GLP1RA, glucagon‐like peptide‐1 receptor agonists; H–L test, Hosmer–Lemeshow test; MedAid, Medical Aid; NHI, National Health Insurance; PVI, Patriots and Veterans Insurance; SGLT2i, sodium‐glucose cotransporter 2 inhibitors.

Regarding GLP1RA, patients > 50 years of age were less likely to use GLP1RA compared to those aged 20–30 years. Female patients were more likely to use GLP1RA (OR = 1.35, [1.06, 1.73]) than male patients. Comorbid dyslipidemia was a positive factor for GLP1RA use. Patients taking statins were more likely to use GLP1RA than those not taking statins. Notably, taking DPP4i emerged as a significant negative factor for GLP1RA use (OR = 0.12, [0.08, 0.16]). Conversely, SU and insulin use were independent strong positive factors associated with GLP1RA use (OR = 3.13, [2.44, 4.02] and OR = 3.71, [2.83, 4.85], respectively). Physicians specializing in cardiology, other internal medicine, and other specialties were less likely to prescribe GLP1RA in comparison to endocrinologists, with cardiologists being least likely to prescribe it (OR = 0.24, [0.10, 0.49]).

### 3.3 Utilization Trends for Each SGLT2i Agent

**FIGURE 3:**
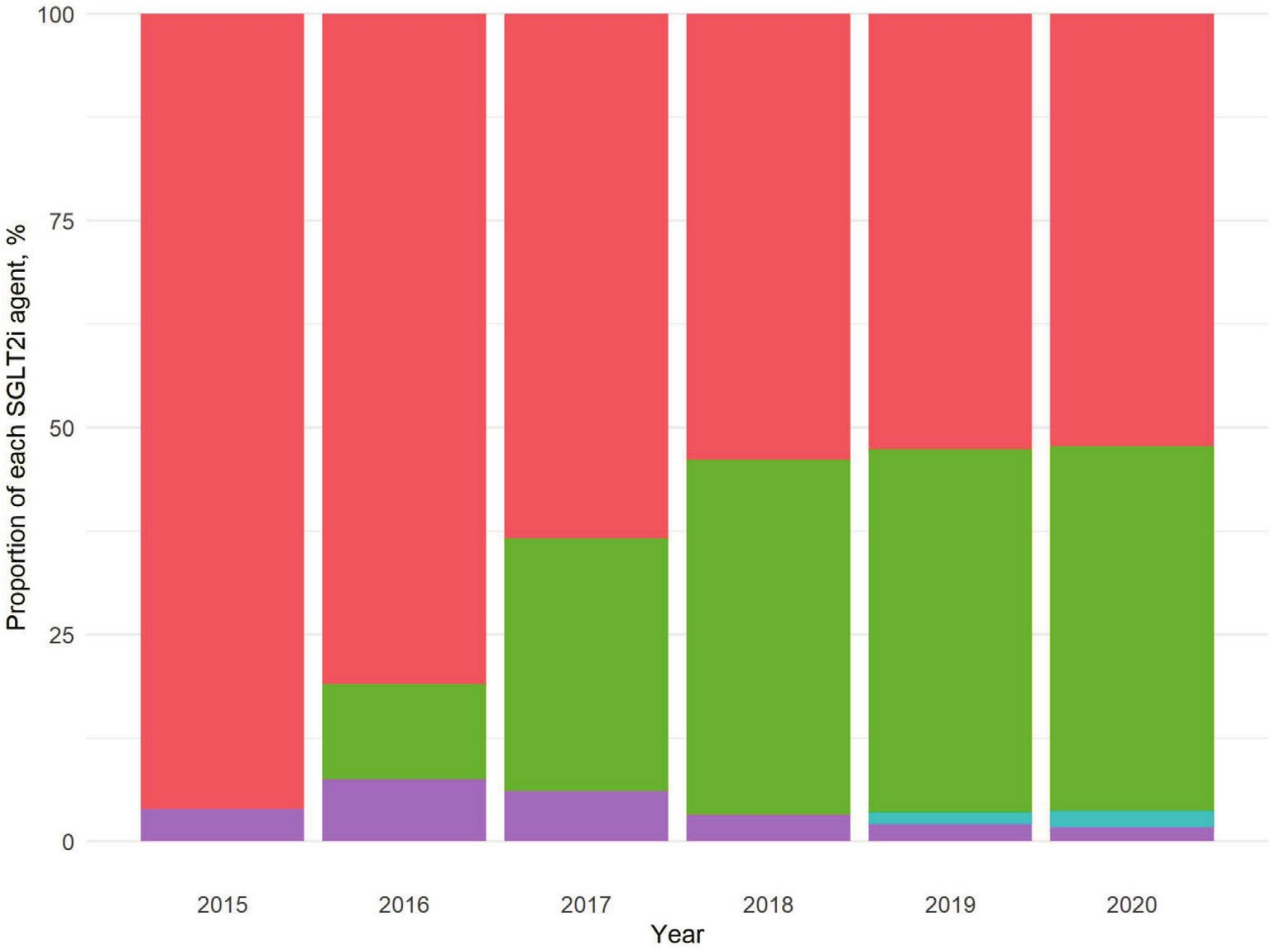
Trends in utilization of each SGLT2i agent between 2015 and 2020. Bars represent the proportion of each SGLT2i agent among total SGLT2i prescriptions: Dapagliflozin (red), empagliflozin (green), ipragliflozin (purple), and ertugliflozin (blue). The Cochran‐Armitage trend test showed a significant trend for all agents (*p* < 0.001), except for ipragliflozin (*p* = 0.463). SGLT2i: Sodium‐glucose cotransporter 2 inhibitors.

Figure 3 illustrates the proportion of each SGLT2i agent in relation to the total SGLT2i utilization per year. Dapagliflozin was dominant in 2015, accounting for 96.08% of SGLT2i use (Table S5). The introduction of empagliflozin in South Korea in 2016 precipitated a consistent upward trend in its share. Conversely, the share of dapagliflozin showed a consistent decline. In 2020, the combined use of dapagliflozin and empagliflozin accounted for more than 96.00% of the total SGLT2i utilization.

### 3.4 Utilization Trends for Each GLP1RA Agent

During the study period, dulaglutide was the most frequently prescribed GLP1RA among patients with T2DM and ASCVD. Its utilization steadily increased over time, surpassing that of exenatide and lixisenatide. The annual prescription trends of individual GLP1RA agents are presented in Table S6.

## 4 Discussion

We observed a gradual increase in the utilization of SGLT2i and GLP1RA in patients with T2DM and ASCVD over the study period. From 2015 to 2020, the utilization rate of SGLT2i increased from 1.20% to 10.51%, whereas the usage rate of GLP1RA increased from 0% to 1.17%. Despite this growth, the utilization of SGLT2i and GLP1RA remained significantly lower than that of medications lacking evidence of cardioprotection, such as DPP4i and SU. Furthermore, the utilization rate of GLP1RA was notably lower than that of any other antidiabetic class.

We found that only 11.65% of eligible patients taking SGLT2i and GLP1RA had received either of these agents by 2020. In contrast to previous studies conducted in the US and Australia, where over 20% of patients with concurrent T2DM and ASCVD were using one of these agent [19, 30], it is evident that a substantial number of patients in South Korea did not receive adequate treatment. Furthermore, the utilization of SGLT2i and GLP1RA was significantly lower than that of noncardioprotective antidiabetic agents. Several previous studies have also shown that despite an increase in the utilization of SGLT2i and GLP1RA, noncardioprotective antidiabetic agents were prescribed more frequently [19, 20, 26]. The underutilization of SGLT2i and GLP1RA could be attributed to clinical inertia. Despite the publication of various clinical trial results and updated guidelines, physicians tend to prescribe older medications over newer recommended options [31, 32]. This tendency may be attributed to a lack of knowledge among physicians, reliance on their own clinical assessments, or consideration of patients preferences [32, 33]. Moreover, the limited use of SGLT2i and GLP1RA could be attributed to stringent insurance coverage criteria and the high costs of these medications. SGLT2i are only covered by the NHI as an add‐on therapy, requiring intolerance to monotherapy or elevated HbA1C levels for eligibility, which restricts their broader use. Similarly, GLP1RA coverage is limited to add‐on therapy status, with eligibility criteria that include intolerance to dual oral agent therapy or insulin. Additional requirements for GLP1RA coverage include BMI of ≥$\;25\mathrm{kg}/m^{2}$ or contraindication to insulin. These strict criteria, combined with the high cost of these newer agents, make them less accessible to patients compared to more established, lower‐cost medications. The extremely low utilization of GLP1RA compared to other countries [19, 23, 24, 25] could be explained by these strict insurance cover requirements. Government efforts to lower the insurance coverage threshold for SGLT2i and GLP1RA are needed to ensure adequate utilization of these agents. In addition, it is important to recognize that the findings of this study reflect prescribing behaviors under the reimbursement criteria applied between 2015 and 2020. During that period, the use of newer agents such as SGLT2i and GLP1RA was limited by strict coverage policies that allowed their use only in specific clinical conditions and restricted combinations. For example, many dual and triple oral agent combinations were not covered, and combinations involving three or more agents were narrowly defined.

However, the reimbursement guideline announced in 2024 expanded the range of reimbursable oral antidiabetic drug combinations. Combinations such as metformin + SGLT2i + sulfonylurea and metformin + SGLT2i + thiazolidinedione, which were previously not explicitly reimbursed, are now clearly supported under the revised policy [34]. These updates represent a shift toward broader, more flexible prescribing options and may help improve patient access to evidence‐based therapies. Therefore, our findings should be interpreted in the context of historical reimbursement restrictions and may not fully reflect current prescribing practices. Future studies using updated datasets are needed to evaluate the impact of these policy changes on real‐world antidiabetic medication use.

We also identified factors influencing the use of SGTL2i and GLP1RA. Factors associated with low SGLT2i use included older age, the presence of comorbid chronic kidney disease, concurrent use of DPP4i and insulin, and receiving prescriptions from other internists and other physicians. Conversely, comorbid dyslipidemia and heart failure, concurrent use of statins and SU, and receiving prescriptions from cardiologists and endocrinologists were associated with high SGLT2i use. Regarding GLP1RA, factors associated with low GLP1RA use included older age, concurrent use of DPP4i, and receiving a prescription from other internists, other physicians, or cardiologists. Conversely, female sex, the presence of dyslipidemia, and concurrent use of statins, insulin, and SU were associated with high GLP1RA utilization.

This study identified lower utilization rates of both SGLT2i and GLP1RA in older patients. This may be attributed to the concerns of physicians regarding complex comorbidities, polypharmacy, and adverse drug reactions (ADR) in this age group [22, 35]. These concerns should be considered; nevertheless, it is essential to emphasize that older patients are more susceptible to cardiovascular diseases [36]. Thus, with closer monitoring and care, prescribing of SGLT2i and GLP1RA should be prioritized in older patients.

Notably, female patients were more likely to use GLP1RA (OR = 1.35, [1.06, 1.73]) than male patients. Weight management is particularly important in individuals with T2DM [37]. GLP1RA not only provides cardiovascular protection but also offers weight loss benefits [38]. Given that weight loss is more pronounced in women than men [38, 39], it is plausible that GLP1RA were prescribed more frequently for weight management in women and were their preferred choice [40]. There was no significant trend to use less SGLT2i for female patients, in contrast to the findings from previous studies [21, 25, 41].

The results also revealed a correlation between prescriptions and comorbidities. Patients with dyslipidemia were more likely to be prescribed both SGLT2i and GLP1RA than those without dyslipidemia. Given that elevated lipid levels are considered a risk factor for cardiovascular disease [42], it is likely that physicians considered the lipid levels of these patients and prescribed SGLT2i and GLP1RA for their cardiovascular benefits. Patients with heart failure were more likely to be prescribed SGLT2i than those without heart failure. While a Canadian study identified concurrent heart failure as a negative factor (OR = 0.77, [0.74, 0.80]) for SGLT2i use in patients with T2DM and ASCVD [21], our study revealed that SGLT2i were appropriately prescribed to patients with heart failure, consistent with the benefits of SGLT2i in heart failure [43]. In contrast, concurrent chronic kidney disease was negatively associated with SGLT2i use but did not affect GLP1RA utilization, despite both classes offering renal benefits [13]. As new indications and clinical evidence for SGLT2i and GLP1RA accumulate, it is essential for physicians to remain informed and assess patient comorbidities before prescribing.

Furthermore, our study identified that the use of DPP4i was significantly negatively associated with both SGLT2i and GLP1RA use (OR = 0.09, [0.09, 0.10] and OR = 0.12, [0.08, 0.16], respectively) in patients with T2DM and ASCVD. Combining DPP4i and GLP1RA is generally not recommended because these two classes of antidiabetic agents share a common mechanism of action, which revolves around enhancing the activity of the incretin hormone GLP‐1, and no additional benefit has been identified when combined [44]. Therefore, this medication combination was not covered by NHI. In contrast, although combining SGLT2i with DPP4i may offer additional advantages [45], such combination therapy was not covered by insurance [26]. This restriction might discourage dual therapies because of concerns about increased patient copayments [46]. Conversely, because combining SU with SGLT2i and GLP1RA was covered by insurance, the use of SU was associated with a higher utilization of both SGLT2i and GLP1RA. Similarly, patients using insulin were more likely to be prescribed GLP1RA. It is important to address concerns regarding the use of SU in patients with ASCVD. Despite their efficacy in glycemic control, SU may not be the most suitable option for patients with existing cardiovascular diseases due to their potential association with increased cardiovascular risk [47, 48]. The cardiovascular risk of SU varies by generation and patient population and is not uniformly observed [49], so caution should be exercised in their use. Nevertheless, the high use of SU in patients with T2DM and ASCVD (36.73% in 2020), along with the positive correlation between SU use and SGLT2i and GLP1RA use, is of great concern. Our findings underscore the need for heightened awareness and education among clinicians to consider cardiovascular outcomes when prescribing antidiabetic medications, particularly for high‐risk populations. Moreover, to ensure that appropriate and comprehensive treatments can be administered according to the patients' clinical conditions, including their cardiovascular risk, government efforts are needed to expand insurance coverage for antidiabetic agent combinations based on the updated clinical guidelines and evidence.

Moreover, differences in the utilization of SGLT2i and GLP1RA were observed based on the physician's specialty. The results of our study suggest an association between cardiologists and increased prescribing of SGLT2i in this particular cohort. Previous studies in other countries have identified lower utilization of SGLT2i by cardiologists than by endocrinologists, along with their limited understanding of the cardiovascular benefits associated with SGLT2i [21, 50]. However, our study demonstrated that cardiologists exhibited a better understanding of the cardiovascular benefits associated with SGLT2i. In contrast, GLP1RA were less likely to be prescribed by cardiologists than endocrinologists (OR = 0.24, [0.10, 0.49]). This could be attributed to patient and cardiologist preferences for oral medications over injectables [51, 52], particularly considering that GLP1RA are only available in an injectable form. Moreover, other internists and physicians were less likely to prescribe both SGLT2i and GLP1RA. Given that this group was responsible for prescribing to more than 60% of patients, there is a clear need to facilitate their prescription of SGLT2i and GLP1RA.

Regarding trends in SGLT2i use, dapagliflozin accounted for 96.08% of total SGLT2i use in 2015. Following the introduction of empagliflozin in 2016, its market share exhibited a consistent upward trend, reaching 44.01% of total SGLT2i use in 2020. This upward trend may have been driven by the publication of trials on empagliflozin and its cardiovascular benefits [53]. The increase between 2018 and 2020 was more gradual than that in previous years, possibly due to the impact of trials related to dapagliflozin published in 2019 [7, 8]. It might have also been influenced by the broader insurance coverage of dapagliflozin in comparison to empagliflozin during the study period. As additional trials continue to demonstrate the cardioprotective effects of other SGLT2i agents and insurance coverage expands, future studies will be necessary to examine changes in the utilization of different SGLT2i components among patients with T2DM and ASCVD.

Our study had some limitations. First, we used claims data collected primarily for reimbursement purposes. Due to the nature of the data, there may be inaccuracies in diagnostic information that differ from the actual clinical information. Additionally, there may be discrepancies between prescribed medications and whether patients actually filled and took them, highlighting the issue of primary nonadherence. While we anticipate that the impact of primary nonadherence is minimal due to the high level of healthcare coverage in South Korea, it could still slightly influence the overall medication filling rates observed in our data. Second, our data lacked clinical information such as plasma glucose level, HbA1C, or disease severity. Furthermore, we lacked socioeconomic data including income levels and education. Although we used disease codes (KCD‐7) instead of clinical parameters to determine patients' conditions, having clinical information would have contributed to a more precise assessment of patients' status and medication use. Similarly, we were unable to consider factors such as patient intolerance, contraindications, or ADR when assessing medication use. Third, GLP1RA with evidence of CVD benefits (liraglutide and semaglutide) were not included in this study due to the lack of insurance cover during the study period. Additionally, prescriptions outside the NHI claims system, such as nonreimbursed or privately covered medications, were not captured in the dataset. However, given the comprehensive nature of Korea's single‐payer health insurance system and the structure of the HIRA‐NPS, we believe the extent of such prescriptions is likely minimal [54]. Fourth, the study data spanned from 2015 to 2020 and did not include more recent years during which clinical guidelines and reimbursement policies have evolved. As such, the findings should be interpreted with caution, particularly in the context of rapidly changing prescribing environments. Given the favorable shifts in the reimbursement and accessibility of both SGLT2i and GLP1RA, there is an increasing need for future research that considers these developments to provide a more comprehensive understanding of their utilization and impact.

Nevertheless, our study provides several significant insights. We have reported recent trends of SGLT2i and GLP1RA in patients with concurrent T2DM and ASCVD. Moreover, we identified not only the characteristics of the patients but also the factors influencing the use of SGLT2i and GLP1RA.

## 5 Conclusions

In patients with T2DM and ASCVD, although the utilization of SGLT2i and GLP1RA increased continually during the study period, a substantial portion of patients (88.35%) did not receive these agents even in 2020. Our analysis suggests disparities in the use of SGLT2i and GLP1RA based on patients' characteristics and physician specialties. Further efforts to explore and address potential barriers to the use of these agents could enhance their clinical benefits by improving access for high‐risk patients.

### 5.1 Plain Language Summary

This study looked at how often newer diabetes medications—SGLT2 inhibitors and GLP‐1 receptor agonists—were used in people with both type 2 diabetes and cardiovascular disease in South Korea. These drugs are known to help prevent heart problems, but the study found that only a small number of patients received them between 2015 and 2020. In fact, by 2020, only about one in 10 eligible patients were prescribed either medication. Older adults and people with other health issues were less likely to be given these drugs. One major reason appears to be the strict insurance coverage rules during that time. Recent changes to Korea's health insurance policies now allow broader use of these medications, which may help more patients access these important treatments in the future.

## Author Contributions

Y.R.E. and N.K.J. conceived and designed the study; Y.R.E., H.J., and S.E.C. performed the analysis; Y.R.E. first drafted the manuscript; each author contributed to drafting the article and endorsed the final version for submission to publication.

## Ethics Statement

The Institutional Review Board of Pusan National University approved this study (PNU IRB/2023_133_HR).

## Consent

The authors have nothing to report.

## Conflicts of Interest

The authors declare no conflicts of interest.

## Supporting information


**Data S1.** Supporting Information.

## Data Availability

HIRA‐NPS data were used in this study and were not permitted to be shared. Raw data were obtained with permission from the Health Insurance Review and Assessment Service of Korea (http://opendata.hira.or.kr).
