# Supplementary material for: Prescribing Patterns of SGLT2 Inhibitors and GLP‐1 Receptor Agonists in Patients With T2DM and ASCVD in South Korea
Source: Pharmacoepidemiol Drug Saf. 2025 Jun 30;34(7):e70183. doi: 10.1002/pds.70183 (PMC12209748; doi:10.1002/pds.70183)
Supplement: Supplementary file 1 — Data S1. Supporting Information. [file PDS-34-e70183-s001.docx]

**Table S1.** Operational definition of atherosclerotic cardiovascular disease (ASCVD) based on KCD-7 diagnosis and procedure codes.

| **Category** | **Disease/procedure type** | **Codes** |
| --- | --- | --- |
| Disease code | Ischemic heart disease | I20, I21, I23, I24, I25 |
|  | Peripheral artery disease | I70, I73, I74 |
|  | Ischemic stroke/Transient ischemic attack | I63, I64, G45.8, G45.9 |
| Procedure code | *Coronary Revascularization* |  |
|  | Percutaneous Transluminal Coronary Angioplasty | M6551, M6552, M6553, M6554 |
|  | Percutaneous Transcatheter Placement of Intracoronary Stent | M6561, M6562, M6563, M6564, M6565, M6566, M6567 |
|  | Percutaneous Transluminal Coronary Atherectomy | M6571, M6572 |
|  | Percutaneous Intravascular Atherectomy | M6620 |
|  | Coronary Angiography | HA670 |
|  | Aortocoronary Venous Bypass Graft Angiography | HA680, HA681, HA682 |
|  | Vascular Bypass Operation (Aorta-Coronary) | O1641, O1642, O1647, OA641, OA642, OA647, O1640, O1648, O1649, OA640, OA648, OA649 |
|  | Percutaneous Thrombus Removal - Thrombolytic treatment (Coronary Artery) | M6634 |
|  | Percutaneous Thrombus Removal - Mechanical Thrombolysis (Coronary Artery) | M6633, M6638 |
|  | Thrombectomy (Artery-Chest) | O2053 |
|  | Angioplasty | OA631, OA634, OA635, OB631, OB634, OB635 |
|  | *Peripheral Artery Revascularization* |  |
|  | Percutaneous Transluminal Angioplasty (Cerebral, Carotid, Aortic, Pulmonary, Others) | M6593, M6594, M6595, M6596, M6597 |
|  | Percutaneous Cerebral Angioplasty with Drug | M6599 |
|  | Percutaneous Intravascular Installation of Metallic Stent (Cerebral, Carotid, Aortic, Pulmonary, Others) | M6601, M6602, M6603, M6604, M6605 |
|  | Percutaneous Intravascular Installation of Stent-Graft (Aortic, Aortic and Iliac, Others) | M6611, M6612, M6613 |
|  | Percutaneous Thrombus Removal - Thrombolytic treatment (Intracranial, Extracranial, Others) | M6630, M6631, M6632, M6635 |
|  | Percutaneous Thrombus Removal - Mechanical Thrombectomy (Intracranial, Extracranial, Others) | M6636, M6637, M6639 |
|  | Vascular Bypass Operation | O0161, O0162, O0163, O0164, O0165, O0166, O0167, O0168, O0169, O0170, O0171, O0172, O0173, O0174, O0175, O0176, O1643, O1644, O1645, O1646 |
|  | Thrombectomy (Artery-Abdomen, Neck, Others) | O2054, O2055, O2056 |
|  | Transluminal Atherectomy (Carotid Artery, Abdominal Artery, Iliac artery, Others) | O0226, O0227, O2064, O2065, O2066, O2067, O2068 |
|  | Angioplasty | OA632, OA633, OA636, OA637, OA638, OA639, OB632, OB633, OB636, OB637, OB638, OB639 |

ASCVD: Atherosclerotic cardiovascular disease; KCD-7: Korean Standard Classification of Diseases, 7th edition.

**Table S2.** Ingredient codes of antidiabetic agents, RAS inhibitors, and statins.

| **Category** | **Medication Class** | **Drug** | **Codes** |
| --- | --- | --- | --- |
| Anti-diabetic medications | Biguanides | Metformin | 191501AT, 191502AT, 191503AT, 191504AT, 191505AT, 421100AT, 443400AT, 443500AT, 452700AT, 452900AT, 469100AT, 471900AT, 474200AT, 474300AT, 498600AT, 497200AT, 498100AT, 498600AT, 502300AT, 502900AT, 507000AT, 507100AT, 513700AT, 524700AT, 519600AT, 518500AT, 518600AT, 518800AT, 520500AT, 520600AT, 520700AT, 523600AT, 523700AT, 523800AT, 631900AT, 632000AT, 632100AT, 635600AT, 635700AT, 637200AT, 639800AT, 641400AT, 641800AT, 641900AT, 642000AT, 644900AT, 645000AT, 648400AT, 648500AT, 648600AT, 649000AT, 649100AT, 649200AT, 649300AT, 649400AT, 649500AT, 649900AT, 650000AT, 650100AT, 653800AT, 653900AT, 654000AT, 654100AT, 655700AT, 671800AT, 673800AT, 671900AT, 672000AT, 672100AT, 672500AT, 672600AT, 672700AT, 672800AT, 672900AT, 673000AT, 675500AT, 683300AT, 683400AT |
|  | Dipeptidyl-peptidase 4 inhibitors | Alogliptin | 624201AT, 624202AT, 624203AT, 630300AT, 630400AT, 630500AT, 630600AT, 675500AT, 635600AT, 635700AT, 675500AT |
|  |  | Anagliptin | 639601AT, 648400AT, 648500AT, 648600AT |
|  |  | Evogliptin | 645301AT, 649900AT, 650000AT, 650100AT |
|  |  | Gemigliptin | 523800AT, 619101AT, 632000AT, 645000AT, 654100AT, 664600AT, 664700AT, 664800AT |
|  |  | Linagliptin | 520500AT, 520600AT, 520700AT, 616401AT |
|  |  | Saxagliptin | 518500AT, 518600AT, 613301AT, 613302AT |
|  |  | Sitagliptin | 501101AT, 501102AT, 501103AT, 502300AT, 502900AT, 513700AT, 524700AT |
|  |  | Teneligliptin | 627301AT, 641800AT, 641900AT, 642000AT |
|  |  | Vildagliptin | 500801AT, 507000AT, 507100AT, 519600AT |
|  | Sulfonylureas | Glibenclamide | 165402AT, 421100AT, 443400AT, 443500AT, 471900AT |
|  |  | Gliclazide | 165602AT, 165603AT, 165604AT, 497200AT |
|  |  | Glimepiride | 165701AT, 165702AT, 165703AT, 165704AT, 474200AT, 474300AT, 498600AT, 525500AT, 525600AT, 488800AT, 488900AT, 489000AT, 498600AT |
|  |  | Glipizide | 165801AT |
|  | Thiazolidinediones | Lobeglitazone | 525901AT, 653800AT, 653900AT, 654000AT, 655700AT |
|  |  | Pioglitazone | 431901AT, 431902AT, 498100AT, 525500AT, 525600AT, 630300AT, 630400AT, 630500AT, 630600AT |
|  |  | Rosiglitazone | 348002AT, 452700AT, 452900AT, 469100AT, 488800AT, 488900AT 489000AT |
|  | Sodium-glucose cotransporter 2 inhibitors | Dapagliflozin | 527301AT, 527302AT, 639800AT, 641400AT |
|  |  | Empagliflozin | 628201AT, 628202AT, 649000AT, 649100AT, 649200AT, 649300AT, 649400AT, 649500AT |
|  |  | Ertugliflozin | 674301AT, 674302AT |
|  |  | Ipragliflozin | 636101AT |
|  | Glucagon-like peptide-1 receptor agonists | Albiglutide | 644501BI, 644502BI |
|  |  | Dulaglutide | 639701BI, 639702BI |
|  |  | Exenatide | 512101BI, 512102BI, 512130BI, 512131BI |
|  |  | Lixisenatide | 626601BI, 626602BI, 626631BI, 626630BI, 666700BI, 667000BI |
|  | Alpha-glucosidase inhibitors | Acarbose | 100602AT, 100601AT |
|  |  | Miglitol | 406201AT, 406202AT |
|  |  | Voglibose | 249001AT, 249002AT, 523600AT, 523700AT |
|  | Meglitinides | Nateglinide | 430201AT, 430202AT, 430203AT, 631900AT, 644900AT |
|  |  | Mitiglinide | 486101AT, 518800AT |
|  |  | Repaglinide | 379501AT, 379502AT, 379503AT, 632100AT, 637200AT |
|  | Insulin | Insulin | 170101BI, 170102BI, 170130BI, 170131BI, 170401BI, 170402BI, 170430BI, 170431BI, 175301BI, 175302BI, 175304BI, 175330BI, 175331BI, 175332BI, 175333BI, 441301BI, 441302BI, 441303BI, 441304BI, 441305BI, 441330BI, 441331BI, 441332BI, 441333BI, 461801BI, 461802BI, 461804BI, 461830BI, 461831BI, 461832BI, 484901BI, 484902BI, 484930BI, 484931BI, 488701BI, 488730BI, 507401BI, 626700BI, 626700BI, 626801BI, 626802BI, 626830BI, 626831BI, 666700BI, 667000BI |
| Angiotensin converting enzyme inhibitors/Angiotensin receptor antagonists | Angiotensin converting enzyme inhibitors | Alacepril | 104201AT, 104202AT |
|  |  | Benazepril | 114701AT |
|  |  | Captopril | 122901AT, 122902AT, 122903AT, 262200AT, 262300AT |
|  |  | Cilazapril | 133001AT, 133002AT, 133003AT, |
|  |  | Enalapril | 151601AT, 151603AT, 440300AT, 453700AT |
|  |  | Fosinopril | 163501AT, 163502AT |
|  |  | Imidapril | 173401AT, 173402AT |
|  |  | Lisinopril | 184501AT, 499200AT, 499300AT |
|  |  | Moexipril | 196801AT, 196802AT |
|  |  | Perindopril | 211301AT, 211302AT, 501601AT, 501602AT, 556200AT |
|  |  | Quinapril | 221901AT |
|  |  | Ramipril | 222401AT, 222402AT, 222404AT, 447100AT, 447200AT, 448600AT, 448700AT |
|  |  | Temocapril | 235002AT |
|  |  | Zofenopril | 510401AT, 510402AT, 510403AT |
|  | Angiotensin receptor antagonists | Azilsartan | 662401AT, 662402AT, 662403AT, 673500AT, 673600AT |
|  |  | Candesartan | 122601AT, 122602AT, 122603AT, 423700AT, 652900AT, 653000AT, 653100AT, 661800AT, 661900AT, 662000AT, 662100AT, 673700AT |
|  |  | Eprosartan | 429201AT, 460500AT |
|  |  | Fimasartan | 515201AT, 515202AT, 515203AT, 522000AT, 526800AT, 651900AT, 652000AT, 652100AT, 652700AT, 654700AT, 654600AT, 654800AT, 654900AT, 655000AT, 684300AT, 684400AT, 684500AT, 684600AT, 684700AT, 688100AT, 688200AT, 688300AT, 688400AT, 688500AT |
|  |  | Irbesartan | 177301AT, 177303AT, 385700AT, 385800AT, 524000AT, 524100AT, 527000AT, 527100AT |
|  |  | Losartan | 185701AT, 185702AT, 262500AT, 378900AT, 486900AT, 502700AT, 503000AT, 513900AT, 637400AT, 637500AT, 637600AT, 662800AT, 662900AT, 663000AT, 663900AT, 664000AT, 664100AT, 664200AT, 664300AT, 664400AT |
|  |  | Olmesartan | 468501AT, 468502AT, 468503AT, 500500AT, 500600AT, 513600AT, 519700AT, 519800AT, 519900AT, 520000AT, 520100AT, 520901AT, 520902AT, 526300AT, 526400AT, 526500AT, 526900AT, 547500AT, 547600AT, 547700AT, 547800AT, 547900AT, 548000AT, 582200AT, 582400AT, 629400AT, 629500AT, 629600AT, 632800AT, 632900AT, 631300AT, 633000AT, 644100AT, 644200AT, 653200AT, 677300AT, 677400AT, 677500AT, 677600AT, 686800AT, 686900AT |
|  |  | Telmisartan | 378801AT, 378802AT, 378803AT, 443200AT, 443300AT, 502600AT, 511500AT, 511600AT, 511700AT, 521200AT, 521300AT, 521400AT, 623100AT, 629900AT, 630000AT, 630100AT, 630200AT, 631600AT, 631700AT, 644800AT, 663500AT, 663600AT, 663700AT, 663800AT, 671200AT, 671300AT, 671400AT, 671500AT, 671600AT, 671700AT, 677000AT, 677100AT, 682700AT, 682800AT, 682900AT |
|  |  | Valsartan | 247101AT, 247102AT, 247103AT, 247104AT, 356400AT, 442600AT, 492800AT, 492900AT, 495800AT, 522200AT, 522300AT, 522400AT, 522600AT, 522700AT, 522800AT, 522900AT, 523000AT, 523100AT, 523200AT, 523300AT, 523400AT, 525000AT, 525100AT, 525200AT, 525300AT, 629700AT, 629800AT, 634900AT, 635000AT, 635100AT, 635200AT, 679500AT, 679600AT, 679700AT, 680300AT, 690400AT, 690500AT, 690600AT, 690700AT, 691400AT, 691500AT |
| Statins |  | Atorvastatin | 111501AT, 111502AT, 111503AT, 111504AT, 472300AT, 472400AT, 472500AT, 502201AT, 502202AT, 502203AT, 502204AT, 518900AT, 524000AT, 524100AT, 527000AT, 527100AT, 614500AT, 633800AT, 633900AT, 634600AT, 634800AT, 671800AT, 673800AT, 671900AT, 672000AT, 672100AT, 688100AT, 688200AT, 688300AT, 688400AT, 688500AT, 690400AT, 690500AT, 690600AT, 690700AT |
|  |  | Fluvastatin | 162401AC, 162402AC, 162403AT |
|  |  | Lovastatin | 185801AT |
|  |  | Pitavastatin | 470901AT, 470902AT, 470903AT, 634900AT, 634900AT, 635000AT, 635100AT, 635200AT, 679300AC |
|  |  | Pravastatin | 216601AT, 216602AT, 216603AT, 216604AT, 519300AC |
|  |  | Simvastatin | 227801AT, 227802AT, 227803AT, 227805AT, 227806AT, 471000AT, 471100AT, 507800AT, 631400AT, 631500AT |
|  |  | Rosuvastatin | 454001AT, 454002AT, 454003AT, 525000AT, 525100AT, 525200AT, 525300AT, 526300AT, 526400AT, 526500AT, 526900AT, 629700AT, 629800AT, 629900AT, 630000AT, 630100AT, 630200AT, 631600AT, 631700AT, 640700AT, 640800AT, 640900AT, 644100AT, 644200AT, 653200AT, 654700AT, 654800AT, 654900AT, 655000AT, 661800AT, 661900AT, 662000AT, 662100AT, 663400AC, 663900AT, 664000AT, 664100AT, 664200AT, 664300AT, 664400AT, 664600AT, 664700AT, 664800AT, 671200AT, 671300AT, 671400AT, 671500AT, 671600AT, 671700AT, 672500AT, 672600AT, 672700AT, 672800AT, 672900AT, 673000AT, 673700AT, 677000AT, 677100AT, 673900AT, 674000AT, 674100AT, 678600AT, 677300AT, 677400AT, 677500AT, 677600AT, 679500AT, 679600AT, 679700AT, 680300AT, 683000AT, 683100AT, 683200AT, 683300AT, 683400AT, 684300AT, 684400AT, 684500AT, 684600AT, 684700AT, 686800AT, 686900AT, 691200AT, 691400AT, 691500AT |

RAS: renin–angiotensin system

**Table S3.** Definition of comorbid conditions.

| **Disease** |  |
| --- | --- |
| Type 2 diabetes mellitus | E11 |
| Hypertension | I10, I11, I13, I12 |
| Dyslipidemia | E78 |
| Heart failure | I50, I110, I130, I132 |
| Chronic kidney disease | N18, N19 |

KCD-7: Korean Standard Classification of Diseases, 7th edition.

**Table S4.** Trends in utilization of antidiabetic drug classes, 2015–2020.

|  | 2015 | 2016 | 2017 | 2018 | 2019 | 2020 |
| --- | --- | --- | --- | --- | --- | --- |
| Total N* | 8 513 | 9 269 | 12 847 | 9 895 | 7 045 | 10 007 |
| Metformin | 6 855 (80.52) | 7 567 (81.64) | 10 492 (81.67) | 7 976 (80.61) | 5 737 (81.43) | 8 302 (82.96) |
| DPP4i | 4 157 (48.83) | 5 338 (57.59) | 7 437 (57.89) | 6 074 (61.38) | 4 278 (60.72) | 6 120 (61.16) |
| SU | 3 885 (45.64) | 3 955 (42.67) | 5 329 (41.48) | 3 961 (40.03) | 2 659 (37.74) | 3 676 (36.73) |
| TZD | 652 (7.66) | 797 (8.60) | 1 240 (9.65) | 958 (9.68) | 679 (9.64) | 1 016 (10.15) |
| SGLT2i | 102 (1.20) | 305 (3.29) | 639 (4.97) | 642 (6.49) | 625 (8.87) | 1 052 (10.51) |
| GLP1RA | 0 (0) | 6 (0.06) | 46 (0.36) | 53 (0.54) | 62 (0.88) | 117 (1.17) |
| Insulin | 1 092 (12.83) | 769 (8.30) | 1 489 (11.59) | 1 225 (12.38) | 929 (13.19) | 1 096 (10.95) |
| AGI | 372 (4.37) | 254 (2.74) | 275 (2.14) | 181 (1.83) | 70 (0.99) | 101 (1.01) |
| MGT | 87 (1.02) | 73 (0.79) | 65 (0.51) | 50 (0.51) | 19 (0.27) | 27 (0.27) |

*Patients with type 2 diabetes mellitus and atherosclerotic cardiovascular disease confirmed between March 1 and October 31 each year who were prescribed at least one anti-diabetic agent.

DPP4i: dipeptidyl-peptidase 4 inhibitors; SU: sulfonylureas; TZD: thiazolidinediones; SGLT2i: sodium-glucose cotransporter 2 inhibitors; GLP1RA: glucagon-like peptide-1 receptor agonists; AGI: alpha-glucosidase inhibitors; MGT: meglitinides.

**Table S5.** Trends in utilization of each SGLT2i agent, 2015–2020.

|  | 2015 | 2016 | 2017 | 2018 | 2019 | 2020 |
| --- | --- | --- | --- | --- | --- | --- |
| Total SGLT2i user | 102 | 305 | 639 | 642 | 625 | 1052 |
| Dapagliflozin | 98 (96.08) | 247 (80.98) | 405 (63.38) | 346 (53.89) | 329 (52.64) | 550 (52.28) |
| Empaglifozin |  | 35 (11.48) | 195 (30.52) | 275 (42.83) | 274 (43.84) | 463 (44.01) |
| Ipragliflozin | 4 (3.92) | 23 (7.54) | 39 (6.10) | 21 (3.27) | 13 (2.08) | 18 (1.71) |
| Ertugliflozin |  |  |  |  | 9 (1.44) | 21 (2.00) |

SGLT2i, sodium-glucose cotransporter 2 inhibitors.

**Table S6.** Trends in utilization of each GLP1RA agent, 2015–2020.

|  | 2015 | 2016 | 2017 | 2018 | 2019 | 2020 |
| --- | --- | --- | --- | --- | --- | --- |
| Total GLP1RA user | 0 | 6 | 46 | 53 | 62 | 117 |
| Dulaglutide | 0 (0) | 2 (33.33) | 38 (82.61) | 50 (93.34) | 47 (75.81) | 98 (83.76) |
| Lixisenatide | 0 (0) | 3 (50.00) | 8 (17.39) | 3 (5.66) | 15 (24.19) | 19 (16.24) |
| Exenatide | 0 (0) | 1 (16.67) | 0 (0) | 0 (0) | 0 (0) | 0 (0) |

GLP1RA: glucagon-like peptide-1 receptor agonists.


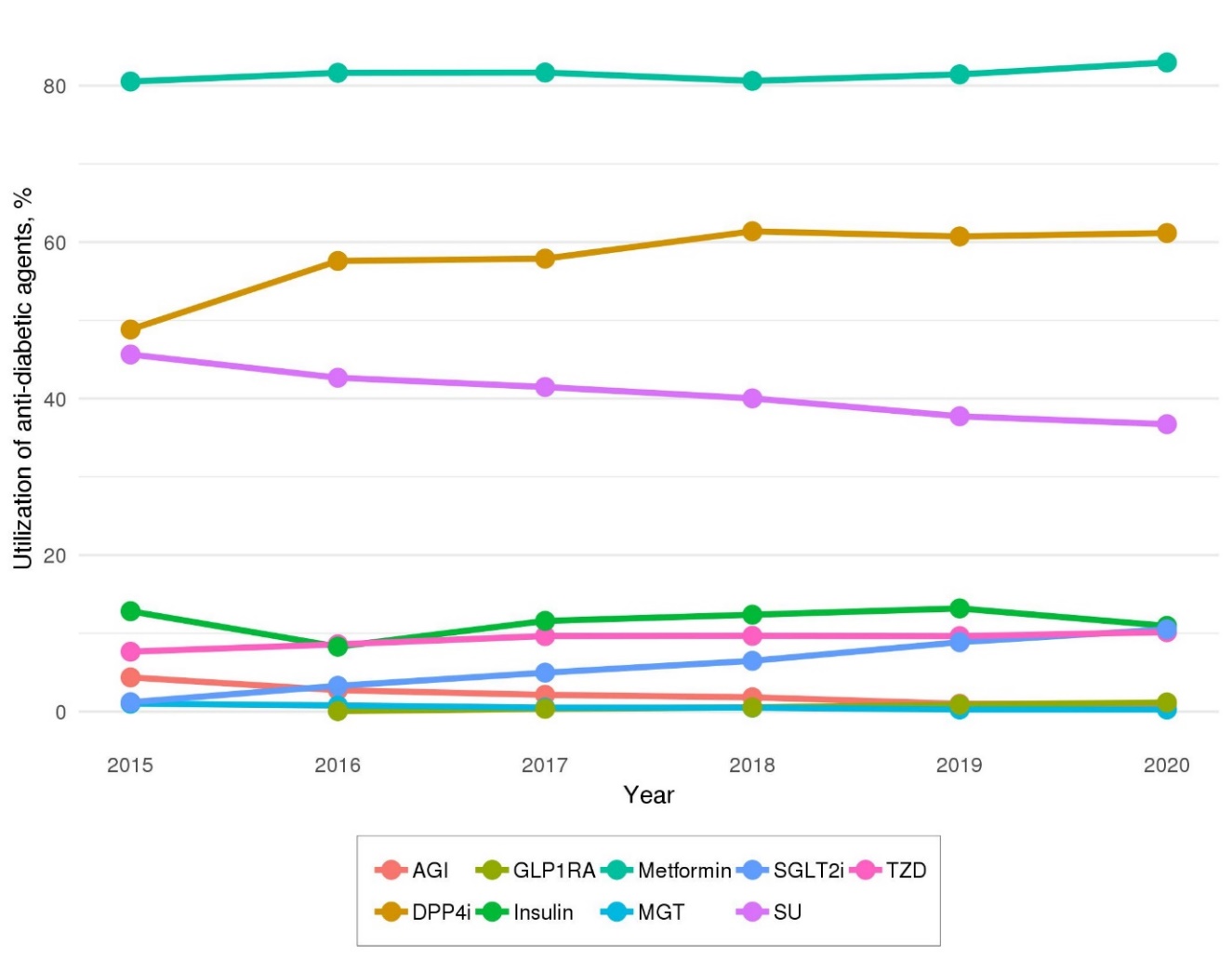


**Figure S1.** Trends in utilization of antidiabetic drug classes, 2015–2020

The *p* value for the Cochran-Armitage trend test of anti-diabetic agents were less than 0.001 (except metformin, insulin and ipragliflozin; metformin: *p* = 0.001, insulin: *p* = 0.567, ipragliflozin: *p* = 0.463).

AGI: alpha-glucosidase inhibitors; SGLT2i: sodium-glucose cotransporter 2 inhibitors; GLP1RA: glucagon-like peptide-1 receptor agonists; DPP4i: dipeptidyl-peptidase 4 inhibitors; SU: sulfonylureas; TZD: thiazolidinediones; MGT: meglitinides.
